# Hematological abnormalities in patients with malaria and typhoid in Tamale Metropolis of Ghana

**DOI:** 10.1186/s13104-018-3456-9

**Published:** 2018-06-05

**Authors:** Nsoh Godwin Anabire, Paul Armah Aryee, Gideon Kofi Helegbe

**Affiliations:** 1 Department of Biochemistry & Molecular Medicine, School of Medicine and Health Sciences, University for Development Studies, P. O. Box TL 1883, Tamale, Ghana; 2 Department of Nutritional Sciences, School of Allied Health Sciences, University for Development Studies, P. O. Box TL 1883, Tamale, Ghana

**Keywords:** Cytopenias, Malaria, Typhoid, Tamale metropolis

## Abstract

**Objective:**

Anemia, Leukopenia, and thrombocytopenia are commonly observed hematological abnormalities in malaria and typhoid patients. In this study, we evaluated the prevalence of cytopenias in patients with mono-infections of plasmodium parasites (malaria group) or salmonella bacteria (typhoid group). Full blood counts from 79 patients (age ranging from 18 to 77 years) categorized into malaria and typhoid groups at the Tamale Central Hospital were assessed.

**Results:**

Data generated were entered and analyzed using SPSS version 20 and Graphpad Prism 6. Values were observed to be significant at p < 0.05. The prevalence of cytopenias were; 29.6, 48.0% for anemia, 38.9, 12.0% for thrombocytopenia, 20.4, 12.0% for leukopenia, 13.0, 8.0% for bicytopenia and 5.6, 4.0% for pancytopenia in both malaria and typhoid groups respectively. Between the two groups of patients, thrombocytopenia was significantly associated with those in the malaria group (*χ*^2^ = 5.84, *p *< 0.016). No association was found between cytopenias and gender in patients in the malaria group; however, the middle aged group, 36–55 years, was significantly associated with anemia (*χ*^2^ = 12.97, *p *< 0.002). Cytopenias were not associated with gender, and with different age categories in patients in the typhoid group.

**Electronic supplementary material:**

The online version of this article (10.1186/s13104-018-3456-9) contains supplementary material, which is available to authorized users.

## Introduction

Cytopenias are blood cell abnormalities that result from reduction in the major hematopoietic cell lines such as red blood cells causing anemia, leukocytes causing leukopenia and thrombocytes causing thrombocytopenia. Bicytopenia occurs where there is a reduction below reference ranges in any two of the major cell lines [1]. The simultaneous presence of anemia, thrombocytopenia and leukopenia in a person is termed pancytopenia.

Anemia [2–5], leukopenia [6–9] and thrombocytopenia [10–16] are commonly presented in *plasmodium* and *salmonella* infections. The occurrence of Cytopenias may be attributed to bone marrow suppression and hemo-phagocytosis [13, 17]. Elsewhere, 94/61.3% anemia, 70/40% thrombocytopenia and 12/4% leukopenia have been reported among adults with malaria or typhoid respectively [14, 18, 19].

Bicytopenia and pancytopenia usually result from direct or indirect decreasing effect on hematopoietic cell production in the bone marrow [20–22]. These kinds of cytopenias are not uncommon in malaria; bone marrow diagnosis of adults with bicytopenia and pancytopenia has shown that 3% of bicytopenia and 6% of pancytopenia were caused by malaria [23, 24]. Even though bone marrow studies have shown no clear explanation for the peripheral blood pancytopenia in typhoid fever [25], a case report of severe pancytopenia in an adult was attributed to hemo-phagocytosis [26].

Despite many global studies reporting anemia, thrombocytopenia and leukopenia with malaria and typhoid in adults, limited studies of these cytopenias exist in Ghana. The studies in Ghana, are mostly on anemia in children [27–30] and in pregnant women [31–34]. Thus, this study was intended to determine the prevalence and association of cytopenias in adults with plasmodium or salmonella infections in Tamale.

## Main text

### Methods

#### Study area

The study was conducted in the Tamale metropolis; the Northern Regional capital of Ghana. The metropolis has 13% of the total land area of the Northern Region (70, 383 square km), and a total population of 223,252 comprising 111,109 males and 112,143 females [35].

#### Study design, study site and population

A hospital based cross-sectional study was conducted from February to April 2015 on patients with mono-infections of plasmodium parasites or salmonella bacteria between the ages of 18–77 years at Tamale central Hospital. Tamale Central Hospital is one of the northern regional hospitals and serves nearly 240,000 people with numerous referrals from other districts in northern Ghana.

#### Sample size and exclusion criteria

Sample size was obtained based on the number of adults that showed up with either malaria or typhoid within the period of sampling. Pregnant women and individuals diagnosed with helminthiasis, sickle cell disease, and kidney disease were excluded from this study.

#### Data collection and processing

Demographic data (including sex and age) of each study participant was collected using a standardized questionnaire (Additional file 1), after consent was obtained from participants. Enumerators were given a one-day training prior to data collection. The questionnaire was pre-tested by random administration to 20 patients. Age was categorized as follows: young adults (18–35), middle aged adults (36–55) and the aged (above 55) [36]. Clinical diagnoses including sickle cell disease, kidney disease, helminthiasis and urine pregnancy tests results were collected from the patients’ folders by the clinicians on duty.

A Vacutainer K_3_EDTA tube (Anhui Kangning Industrial group, Tianchang, China, Catologue Number: VG4000515) was used to collect 3 ml of venous blood from each participant for laboratory investigation. The blood samples were collected by a trained medical laboratory scientist.

Malaria was diagnosed using CareStart™ HRP-2 rapid diagnostic test (Access Bio Inc., New Jersey, USA, Catalogue number: G0141). The testing, results reading and interpretations were performed by strictly following the manufacturer’s protocol. The presence of malaria parasites was confirmed by microscopy using thick blood smears which were stained with 20% Giemsa solution at pH 7.2 [37]. The reading of the blood smears and interpretation of the results were done as previously reported [38].

The slide agglutination method previously described [39] was performed for typhoid using commercially available Widal kits (Lab Care Diagnostics, Mumbai, India, Catalogue number: CW0111). Briefly, patients’ plasma was titrated in the following volumes 40, 20, 10 and 5 μl. One plate was designated for antigen-O and the other antigen-H. Later, one drop of antigen-O and antigen-H of *Salmonella typhi* were added to patient’s plasma distributed on the respective plates. This was then mixed and rocked and agglutination observed and interpreted as previously reported [39].

Hb-Hemoglobin, WBC-White blood cell and PLT- Platelets were analyzed using the automated blood cell analyzer Sysmex XS-500i (Sysmex Corporation, Kobe, Japan). Anaemia was diagnosed by Hb < 12 g/dl for non-pregnant females and Hb < 13 g/dl for males [40], while PLT count < 150 × 10^9^/l and WBC count < 4.0 × 10^9^/l were used respectively to determine thrombocytopenia and leukopenia [41]. Bicytopenia was determined if a subject had a combination of any 2 cytopenias and pancytopenia as simultaneously having all three forms of cytopenia.

#### Statistical analysis

Data were analyzed using SPSS Version 20 (IBM Corporation, Chicago, USA) and Graphpad Prism 6 (GraphPad Software Inc., San Diego, USA). All test statistics with p values < 0.05 were considered as statistically significant. Continuous variables were described using median and interquartile range (IQR), and compared by Mann–Whitney test, whilst categorical variables were presented as counts and percentages, and compared by Fisher’s exact test or Pearson’s *χ*^2^ test.

### Results

#### General characteristics of the study participants

Out of the 79 individuals recruited for this study, majority, 55 (69.6%) were females (Table 1). The median age was 31 years (IQR 21–47 years) with majority of the study participants, 47 (59.5%) being young adults (18–35 years), Table 1. The median hemoglobin count was 12.8 g/dl (IQR 11.3–14.1 g/dl), whilst the median platelet and white cell counts were 213 × 10^3^/µL (IQR 142.0–287 × 10^3^/µL) and 5.8 × 10^3^/µL (IQR 4.5–7.0 × 10^3^/µL) respectively.

**Table 1:** Socio-demographic characteristics and hematological profile among malaria and typhoid patients

Characteristics	Malaria group (*N* = 54)	Typhoid group (*N* = 25)	*p* value* (95% CI)
Age, years, median (IQR)	28 (20–45)	37 (24–54)	0.1544 (− 2.000 to 14.000)
Age category, years, *n* (%)			
18–35	36 (66.7)	11 (44.0)	0.069 (0.371 to 1.530)
36–55	9 (16.7)	10 (40.0)	
56+	9 (16.7)	4 (16.0)	
Gender, *n* (%)			
Male	14 (25.9)	10 (40.0)	0.206 (0.192 to 1.435)
Female	40 (74.1)	15 (60.0)	
Hemoglobin, median (IQR)	13.3 (11.8–14.3)	12.6 (11.1–13.3)	0.0986 (− 1.800 to 0.200)
Platelets, median (IQR)	197.5 (120.5–273.5)	284.0 (175.5–320.0)	*0.0065 (20.00 to 118.00)*
White blood cells, median (IQR)	5.7 (4.3–6.6)	6.2 (4.9–9.8)	0.2301 (− 0.410 to 1.760)
Anemia, *n* (%)	16 (29.6)	12 (48.0)	0.112 (− 0.431 to 0.411)
Thrombocytopenia, *n* (%)	21 (38.9)	3 (12.0)	*0.016 (1.241 to 17.550)*
Leukopenia, *n* (%)	11 (20.4)	3 (12.0)	0.530 (0.474 to 7.430)
Bicytopenia, *n* (%)	7 (13.0)	2 (8.0)	0.711(0.392 to 8.911)
Pancytopenia, *n* (%)	3 (5.6)	1 (4.0)	1.000 (0.139 to 14.300)

Source: Field Survey. *Analyzed using Mann–Whitney test and Pearson’s *χ*^2^ test or Fisher’s exact test. Two tailed *p* value significant at *p* < 0.05

*Malaria group* patients with plasmodium mono-infection, *Typhoid group* patients with salmonella mono-infection

#### Prevalence and association of cytopenias in patients with malaria or typhoid

The prevalence of cytopenias were; 29.6, 48.0% for anemia, 38.9, 12.0% for thrombocytopenia, 20.4, 12.0% for leukopenia, 13.0, 8.0% for bicytopenia and 5.6, 4.0% for pancytopenia in patients with mono-infections of *plasmodium* and *salmonella* respectively (Table 1). While thrombocytopenia was significantly associated (*p *< 0.016), with patients in the malaria group, anaemia, leukopenia, bicytopenia and pancytopenia were independent of either groups (Table 1). Similar levels of Hb and leucocytes were observed between both groups, however, levels of platelets were statistically lower, *p *= 0.007 in the malaria group compared to the typhoid group (Table 1). Pancytopenia was only found in females (7.5% in malaria and 6.7% in typhoid), Table 2. Cytopenias were observed to be independent of gender and most age categories for either groups, however, the middle aged malaria group, was significantly associated with anemia (*p *= 0.002), Table 2.

**Table 2:** Association between cytopenias; and gender and age groups in malaria (N = 54) and typhoid patients (N = 25)

Characteristics	^α^Malaria group	^β^Typhoid group	^α^Malaria group	^β^Typhoid group	*p* value* (95% CI)
	Anemia				
	Yes, *n* (%)		No, *n* (%)		
Male	4 (28.6)	6 (60.0)	10 (71.4)	4 (40.0)	^α^1.000 (0.244–3.574), ^β^0.428 (0.439–11.530)
Female	12 (30.0)	6 (40.0)	28 (70.0)	9 (60.0)	
18–35	6 (16.7)	6 (54.5)	30 (83.3)	5 (45.5)	^α^*0.002 (0.009–0.346)*, ^β^0.798 (0.318–10.210)
36–55	7 (77.8)	4 (40.0)	2 (22.2)	6 (60.0)	
56+	3 (33.3)	2 (50.0)	6 (66.7)	2 (50.0)	
	Thrombocytopenia				
	Yes, *n* (%)		No, *n* (%)		
Male	6 (42.9)	0 (0.0)	8 (57.1)	10 (100.0)	^α^0.723 (0.363–4.308), ^β^0.051 (0.003–1.409)
Female	15 (37.5)	6 (40.0)	25 (62.5)	9 (60.0)	
18–35	14 (38.9)	3 (27.3)	22 (61.1)	8 (72.7)	^α^0.349 (0.027–12.300), ^β^0.114 (0.390–3.772)
36–55	2 (22.2)	0 (0.0)	7 (77.8)	10 (100.0)	
56+	5 (55.6)	0 (0.0)	4 (44.4)	4 (100.0)	
	Leukopenia				
	Yes, *n* (%)		No, *n* (%)		
Male	2 (14.3)	1 (10.0)	12 (85.7)	9 (90.0)	^α^0.708 (0.108–3.053), ^β^0.179 (0.017–1.680)
Female	9 (22.5)	6 (40.0)	31 (77.5)	9 (60.0)	
18–35	8 (22.2)	2 (18.2)	28 (77.8)	9 (81.8)	^α^0.752 (0.248–21.110), ^β^0.612 (0.153–3.360)
36–55	2 (22.2)	1 (10.0)	7 (77.8)	9 (90.0)	
56+	1 (11.1)	0 (0.0)	8 (88.9)	4 (100.0)	
	Bicytopenia				
	Yes, *n* (%)		No, *n* (%)		
Male	3 (21.4)	1 (10.0)	11 (78.6)	9 (90.0)	^α^0.358 (0.475–12.690), ^β^1.000 (0.106–12.460)
Female	4 (10.0)	1 (6.7)	36 (90.0)	14 (93.3)	
18–35	2 (5.6)	1 (9.1)	34 (94.4)	10 (90.9)	^α^0.057 (0.016–0.860), ^β^0.811(0.048–2.076)
36–55	2 (22.2)	1 (10.0)	7 (77.8)	9 (90.0)	
56+	3 (33.3)	0 (0.0)	6 (66.7)	4 (100.0)	
	Pancytopenia				
	Yes, *n* (%)		No, *n* (%)		
Male	0 (0.0)	0 (0.0)	14 (100.0)	10 (100.0)	^α^0.560 (0.018–7.611), ^β^1.000 (0.017–12.460)
Female	3 (7.5)	1 (6.7)	37 (92.5)	14 (93.3)	
18–35	2 (5.6)	1 (9.1)	34 (94.4)	10 (90.9)	^α^0.589 (0.038–5.858), ^β^0.811(0.048–2.076)
36–55	1(11.1)	1 (10.0)	8 (88.9)	9 (90.0)	
56+	0 (0.0)	0 (0.0)	9 (100.0)	4 (100.0)	

Source: Field Survey. *Analyzed using Pearson’s *χ*^2^ test or Fisher’s exact test, ^α^and ^β^represent *p* values for association of cytopenias with gender and with age groups in Malaria group: patients with plasmodium mono-infection, and Typhoid group: patients with salmonella mono-infection, respectively. Two tailed *p* value significant at *p* < 0.05. 95% CI 95% confidence interval

### Discussion

Malaria and typhoid are quite common in Tamale because the prevailing warm and humid climate is conducive for the infection, concomitant with poor sanitary practices that promote the infections [42]. Malaria endemicity is nearly the same across the year but peaks slightly in the rainy season [43]. Despite the frequent diagnoses of anemia, thrombocytopenia and leukopenia in patients presenting with malaria or typhoid at the health facilities, not much is known about the prevalence of these cytopenias among adults in Ghana; most especially in northern Ghana. Thus, providing the impetus for this study in Tamale. To the best of our knowledge, we are, for the first time, reporting the prevalence of cytopenias in patients with malaria or typhoid in Ghana.

In the current study, thrombocytopenia was found to be the most prevalent in the adults with plasmodium infection. This contradicts 2 studies [18, 19] that showed anemia as the most prevalent cytopenia in malaria patients. However, both studies were conducted in south Asia where the dominant malaria parasite (*P. vivax*) is different from that in Ghana (*P. falciparum*). Unlike *P. vivax*, *P. falciparum* is very virulent; it multiples rapidly in the blood and has the ability to sequester into diverse host organs [44]. It has been shown that some *P. falciparum* infected-erythrocytes can bind platelets to form platelet-mediated clumps [45] and postulations are that immune mediated lysis of these clumps cause thrombocytopenia in *P. falciparum* infection. Thus, differences in parasite strains could be a possible reason for the difference seen in this study; more so because [19] realized a significant increase in the incidence of thrombocytopenia in *P. falciparum* cases than in *P. vivax* cases. Our findings of a higher prevalence and significant association of thrombocytopenia with malaria adds to studies indicating that thrombocytopenia is an indicator of malaria in the adult population [46].

While extensive studies have been conducted on the occurrence of anemia in children and pregnant women with malaria [2–5], little is known of such studies in non-pregnant female and male adults. In areas with high malaria transmission, adults usually develop semi-immunity to malaria due to exposure to several different clones of malaria parasites. As such, adults are mostly asymptomatic, with low parasite loads that could lead to anemia and its related complications. In this study, anemia was strongly associated with the middle aged group, 35–55 years but was, however, gender independent. Even though it has been shown that anemia in malaria patients differ in different ages [17], it is not clear why anemia is significantly associated with this particular age group. Nevertheless, it is tempting to speculate that immune reaction is very active in this age group resulting in high loss of uninfected RBCs as well as the infected ones [47]. This should prompt a closer look at anemia with malaria infection in this age category in Tamale.

Even though leukopenia was the least prevalent cytopenia, its prevalence was quite significant, and could confound the estimation of malaria parasite density in adults in Tamale based on the WHO criteria of WBC count of 8000 cells/μL.

The high prevalence of anemia recorded in the adults with typhoid is similar to a study by [14]. This finding could be attributed to myeloid maturation arrest and decrease in the number of erythroblasts as shown by [13]. The independence of the cytopenias with typhoid, age and gender among the study population may suggest that Hb, PLT and WBCs counts may not be good hematological markers for the diagnosis of typhoid in adults in Tamale.

Pancytopenia and bicytopenia are a common hematological problem encountered in clinical practice, which have multiple causes and the underlying pathology determines the management and prognosis of the patients. Bicytopenia and pancytopenia were observed in both infections; and the knowledge that both cytopenias are seen in quite a number of malaria and typhoid patients in Tamale is important information that would help in timely diagnosis and proper management of such cases, leading to earlier discharge of the patient. Unlike many studies that had male dominance in pancytopenia cases [48–51], we realized that pancytopenia was only found in females for either infection, which could possibly be attributed to the female dominance in this study. This falls in line with what pertains in Ghana, where women are more likely to access health care. More to the point, women often have to ask for permission from their husbands in order to access treatment for themselves, which may delay depending on whether their husbands or family elders would agree [52]. This may explain why in this study, the worse form of cytopenia, pancytopenia is only found in females, because the longer it takes for one to get an appropriate treatment for these infections, the more damage the disease causes to one’s hematopoietic system.

### Conclusion

The high prevalence and association of thrombocytopenia with malaria in adults in Tamale buttresses numerous findings of low platelets count as a reliable diagnostic marker for malaria in adult populations in Africa. The association of anemia with the middle aged adults with malaria should prompt a closer look at anemia with plasmodium infection in this age category in Tamale. Our finding of pancytopenia in only females, should prompt strengthening of education on barriers preventing females from accessing healthcare delivery.

## Limitation

This study was conducted within a relatively shorter period and the sample size was small. Since malaria endemicity is nearly constant within the metropolis, an all year investigation of these cytopenias in patients with malaria or typhoid would be important in substantiating the findings in this study.

## Additional files

**Additional file 1.** Document used in the collection of socio-demographic and health data. This is a blank document which contains the questionnaire used in the collection of socio-demographic characteristics, and the health records of the study participants.

**Additional file 2.** Data set of the subjects. This is a SPSS document containing data transribed from the questionnaire.

## Abbreviations

Hb: hemoglobin
WBCs: white blood cells
PLT: platelets
IQR: interquartile range
RBCs: red blood cells
WHO: World Health Organization
