# Supplementary material for: Hematological abnormalities in patients with malaria and typhoid in Tamale Metropolis of Ghana
Source: BMC Res Notes. 2018 Jun 5;11:353. doi: 10.1186/s13104-018-3456-9 (PMC5989466; doi:10.1186/s13104-018-3456-9)
Supplement: Supplementary file 1 — Additional file 1. Document used in the collection of socio-demographic and health data. This is a blank document which contains the questionnaire used in the collection of socio-demographic characteristics, and the health records of the study participants. [file 13104_2018_3456_MOESM1_ESM.docx]

**University for Development Studies**

**School of Medicine and Health Sciences (SMHS)**

**Department of Biochemistry and Molecular Medicine Survey Questionnaire**

**_____________________________________________________**

**Investigation of hematological abnormalities in patients with malaria and typhoid in Tamale Metropolis of Ghana**

**Consent for Questionnaire administration**

Good morning/afternoon/evening. My name is Nsoh Godwin, a Research Assistant at the Department of Biochemistry and Molecular Medicine, University for Development Studies, Tamale, Ghana. I am conducting a study on the topic ‘**Investigation of hematological abnormalities in patients with malaria and typhoid in Tamale Metropolis of Ghana’**. I would like to have an interview with you and would very much appreciate your participation. This interview will take about 10 minutes to complete. All of the answers you will provide will be confidential and will not be seen by anyone apart from me. If I should come to any question you don’t want to answer, just let me know and I will go on to the next question. However, I hope you will participate fully in the survey since your views are important. May we begin the interview now?

1. Yes 2. No

Questionnaire number…………………CODE FOR LABELING SAMPLE……………………

**SECTION A: SOCIO-DEMOGRPHIC FACTORS**

A1. Age of respondent: ………………. Yrs

A2. Sex of respondent: (1) Male (2) Female

A3. What is your religion?

(1). Christianity (2).Islam (3).ATR (4).Others…………………….

A4. Ethnicity…………………

A5. What is your level of education?

(1) No education (2) Basic education (3) Secondary education (4) Tertiary education

A6. Occupation of respondent

(1) Teacher (2) Trader (3) Farmer (4) Student (5) Others………………………

A7. Marital status of respondent.

(1) Married (2) Single (3) Separated (4) Divorced (5) Widow/Widower

A8. How do you evaluate your financial situation?

(1) Very poor (2) Poor (3) Good (4) Very good

***THANK YOU!!!***

**SECTION B: HEALTH RECORDS**

1. Helminthiasis: Yes……………… No ………………..
2. Sickling status: Negative…………… Positive ………………
3. Kidney disease: Yes ……………….. No………………
4. Hepatitis B status: Positive ….…………… Negative………………
5. Hepatitis C status: Positive ……………. Negative
6. HIV status: Positive……………….. Negative………………
7. Syphilis status: Positive…………….. Negative……………
8. Urine pregnancy test: Positive……….. Negative………

***THANK YOU!!!***
